# The components of the *Daphnia pulex *immune system as revealed by complete genome sequencing

**DOI:** 10.1186/1471-2164-10-175

**Published:** 2009-04-22

**Authors:** Seanna J McTaggart, Claire Conlon, John K Colbourne, Mark L Blaxter, Tom J Little

**Affiliations:** 1 Institute of Evolutionary Biology, University of Edinburgh, Edinburgh, UK; 2 Center for Genomics and Bioinformatics, Indiana University, Bloomington, USA

## Abstract

**Background:**

Branchiopod crustaceans in the genus *Daphnia *are key model organisms for investigating interactions between genes and the environment. One major theme of research on *Daphnia *species has been the evolution of resistance to pathogens and parasites, but lack of knowledge of the *Daphnia *immune system has limited the study of immune responses. Here we provide a survey of the immune-related genome of *D. pulex*, derived from the newly completed genome sequence. Genes likely to be involved in innate immune responses were identified by comparison to homologues from other arthropods. For each candidate, the gene model was refined, and we conducted an analysis of sequence divergence from homologues from other taxa.

**Results and conclusion:**

We found that some immune pathways, in particular the TOLL pathway, are fairly well conserved between insects and *Daphnia*, while other elements, in particular antimicrobial peptides, could not be recovered from the genome sequence. We also found considerable variation in gene family copy number when comparing *Daphnia *to insects and present phylogenetic analyses to shed light on the evolution of a range of conserved immune gene families.

## Background

All metazoans appear to have an innate immune system based on a distinct set of gene products that play recognition, regulatory and response roles [1]. However, within this set of genes, there is striking diversity, as the enormous spectra of host habitats and specialist biological enemies drive the evolution of immune systems [2-6]. To date, knowledge of immune system function in arthropods has been based on a very few model organisms, and the majority of studies have used two members of a single insect order, specifically the Dipterans *Drosophila melanogaster *and *Anopheles gambiae*. While the genes associated with immunity are now being characterised in other insect genomes [7,8], these additional exemplars do not yet span the diversity of Arthropoda. To gain fuller understanding of both the evolutionary origins of the insect immune system, and of any underlying patterns in arthropod immune system function and diversification, it is necessary to examine species in other arthropod subphyla, such as the Crustacea.

*Daphnia *(waterfleas, Family Daphniidae, Order Branchiopoda) species are employed as model organisms for a wide range of evolutionary and ecological topics, including the evolution of immunity, coevolution and virulence [4,9-14]. *Daphnia *and many of their parasites can be manipulated experimentally, and laboratory experiments have revealed a wealth of genetic diversity for responses to infection [9]. Especially useful is the fact that *Daphnia *are cyclical parthenogens, and thus can be maintained in the lab as clonal lineages, enabling a precise comparison of phenotypes between different genetic backgrounds, or the study of different environments on the same genetic background. As they can also reproduce sexually, traditional crossing experiments are feasible. Daphnia also provide an unprecedented opportunity to elucidate the natural variation in immune responses over long timescales (decades to centuries) [15-17]. In natural habitats, Daphnia produce diapausing resting eggs, most of which are buried in the sediments, where they remain viable for up to 200 years [17]. Through the use of dated sediment cores, the past record of evolutionary change can be resurrected by hatching these eggs. The molecular record of evolution can be extended even further, as DNA can be acquired from resting eggs up to 3000 years old [16].

While it has been easy to identify and measure the phenotypic consequences of variation in infection outcomes, the molecular and physiological mechanisms underpinning this variation have not been accessible in *Daphnia*. In contrast, the genetics of immune function have been characterised in detail in more traditional models such as *D. melanogaster*. Genes of arthropod defence can be grouped into five main functional classes [18-21]: (1) pathogen recognition, (2) signal modulation, (3) signal transduction, (4) attack, and (5) anti-viral RNA interference.

Recognition receptors (called pattern recognition receptors; PRR) detect pathogen-associated molecular patterns (typically conserved cell-surface motifs), and signal this to modulating signalling receptors. These receptors in turn initiate cascades that result in transcriptional and translational activation of attack molecules. Some effectors are activated by post-translational proteolytic cleavage. Three cascades are particularly well studied. First, the Toll pathway is activated when fungal and bacterial products stimulate transmembrane Toll receptors, which ultimately lead to the production of antimicrobial peptides, melanization through prophenoloxidase or proteasome-dependent degradation. Secondly, the Imd pathway, induced when bacterial or fungal products are detected by peptidoglycan-LC type receptors, can also lead to the production of antimicrobial peptides, melanization or apoptosis. Finally, the Jak/Stat pathway results in the production of thioester containing proteins (TEPs), which can bind pathogens until they are cleared through phagocytosis. The RNAi pathway acts intracellularly to recognise, process and finally destructively cleave specific double-stranded RNAs that likely derive from viral infection.

A comparison of immune genes from the first four of these functional classes from two species of mosquito, *A. gambiae*, and *Aedes aegypti*, and the fruit fly *D. melanogaster *revealed that the number of orthologous trios and their sequence divergence differs among functional classes, confirming that genes from different classes are under different selective pressures [22]. Specifically, it was observed that genes in the signal transduction pathways have strict 1:1:1 orthologues, and their sequences are more divergent than orthologous trios from the other three classes. In contrast, the effector molecules have very few orthologous trios with lower sequence divergence than gene trios from the other classes. The evolutionary signature of the other functional classes falls between these extremes.

Using prior data on invertebrate immune system genes, we have searched the recently produced draft genome sequence of the waterflea *Daphnia pulex *to identify homologues of genes with demonstrated immune function in other arthropods. By carefully refining the gene models for these putative immune genes we have produced revised protein predictions, and here present an analysis of both the diversity of the *D. pulex *immune genome and its relation to those of other arthropods.

## Results and discussion

### Overview

We identified and annotated 82 genes representing 21 unique gene families from the *Daphnia pulex *v1.1 draft genome sequence assembly (September, 2006) (Table 1) [23-26]. In parallel, we collated information from the genome sequences of five insect species to identify changes in gene family membership among taxa. For the phylogenetic analyses, we included additional arthropod sequences when they were available. The differences in gene family membership among taxa are of particular interest as they may reflect the evolutionary genomic response to the unique repertoire of immune challenges that a species has faced, and thus provide clues as to which genes are evolving in response to host/parasite interactions. For example, recognition and effector molecules that interact directly with pathogens display considerable species-specific gene expansion, in contrast to signal transduction molecules, which show no copy number variation and high sequence divergence [22].

**Table 1:** Annotated gene copy number for five species of insects and the crustacean *Daphnia pulex*

**Protein family**	***Dpul***	***Agam***	***Aaeg***	***Dmel***	***Tcas***	***Amel***
*Recognition*						
PGRP	0	7	8	13	6	4
TEP	7	13	8	6	4	4
GNBP	11	7	7	3	3	2
Scavenger A	6	5	5	5	4	3
C-type-lectin	6	25	39	34	16	10
Galectin	3	10	12	5	3	2
*Transduction*						
Toll/Toll related	7	10	12	9	9	5
Relish	1	2	3	3	2	2
MyD88	1	1	1	1	1	1
Pelle	1	1	1	1	1	1
Tube	0	1	1	1	1	1
Cactus	1	1	1	1	1	3
Imd	1	1	1	1	1	1
STAT	1	2	1	1	1	1
*Attack*						
Chitinase	17	13	19	16	16	5
Prophenoloxidase	1	9	10	3	3	1
Caspase	8	15	10	8	8	1
Nitric oxide synthase	2	1	1	1	1	1
*Others*						
Argonaute	2	2	2	2	2	2
Dicer	3	2	2	2	2	1*
DSCAM	1	1	1	1	NK	1
Gemini	1	NK	NK	1	NK	1
Dorsal	1	1	2	1	NK	2

* two copies of dicer seemed likely for *A. mellifera*, but could not be confirmed.

Genes are classified according to function and displays the current number of annotated gene copies of each gene in the waterflea *D. pulex *(Dpul), the mosquitoes *Anopheles gambiae *(Agam) and *Aedes aegypti *(Aaeg), the fruit fly *Drosophila melanogaster *(Dmel), the flour beetle *Tribolium castaneum *(Tcas), and the honey bee *Apis mellifera *(Amel). Gene counts were gathered from [7,8,22] and public genome databases [24-26]. Please note that gene copy number may differ slightly among sources. NK indicates that the gene is not annotated from that taxon. Sequences of all of the *Daphnia pulex *gene models are available in Additional File 5.

Overall, our search for immune system homologues uncovered fewer genes in *D. pulex *than in *D. melanogaster, A. gambiae *or *A. aegypti*, a similar repertoire to *T. castaneum*, and more genes than *A. mellifera *(Table 1). The fact that we found fewer genes than are present in the dipteran genomes may be an artefact due to the high degree of sequence divergence between query and target sequences. However, *D. pulex *gene families are not consistently lower in number in comparison to the three dipteran species. For example, *D. pulex *has 11 members of the gram-negative bacterium binding protein (GNBP) family, while *D. melanogaster *has 3 members, and *A. gambiae *and *A. aegypti *each have 6 members. Thus, lower copy numbers in *D. pulex *are unlikely to be entirely due to the use of dipteran sequences to search a crustacean genome, and may instead reflect real differences in these organisms' evolutionary histories and subsequent strategies in combating their respective pathogens.

### Gram-negative binding proteins (GNBPs)

GNBPs are PRR that bind pathogens involved in initiating the prophenoloxidase and Toll immune system cascades. There are two distinct groups of GNBPs, characterised by the presence or absence of the cysteine rich (CR) domain, which binds compounds of pathogenic origin (e.g. β-1-3-glucan, lipopolysaccharide, or lipotechoic acid). All of the GNBP genes of *D. melanogaster*, *A. mellifera *and *Bombyx mori *have the CR domain. In contrast only two of seven GNBP genes of *A. gambiae *contain this domain. Additionally, all GNBPs, except two from *D. melanogaster *(CG13422 and CG12780), contain a glucanase-like (GLU) domain that is susceptible to protease digestion and has lower affinity for polysaccharides than the CR domain. In all crustaceans examined previously the GLU domain contains a putative catalytic site that is absent in *D. melanogaster*. *A. gambiae *and *D. pulex *have genes both with and without the putative catalytic site.

Eleven *D. pulex *GNBP genes were found. Three scaffolds contain two GNBP genes each whilst one scaffold has four GNBP genes, three of which are in close proximity to each other. Dappu-GNBP2 is alone on a separate scaffold. The number of exons in the *D. pulex *GNBP genes is typically six or seven but Dappu-GNBP2 has nine exons. Overall, the conservation of intron/exon boundaries is apparent and may indicate recent duplication events, a model supported by phylogenetic analysis suggesting that *D. pulex *GNBP family expansion is recent on an evolutionary timescale.

**Figure 1:**
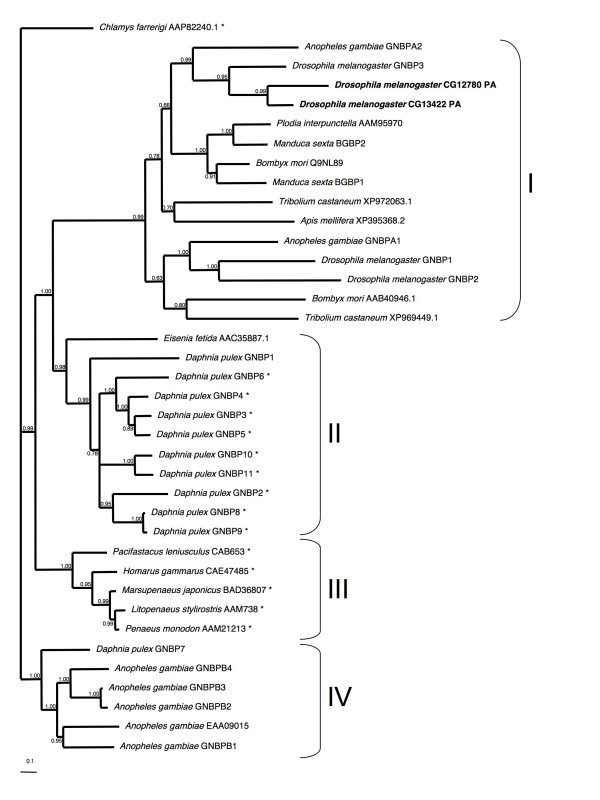
**Bayesian phylogenetic analysis of the Gram-negative binding proteins (GNBPs) from available insect and crustacean species**. All genes containing a catalytic site in their glucanase-like domain are marked with asterisks (*). All GNBP genes known to be involved in the immune system are highlighted in bold text. Branch numbers are posterior probabilities.

Phylogenetic analysis shows that the GNBP fall into four well-supported clades (Figure 1). GNBP clade I is insect-specific, and contains proteins with CR domains and inactive GLU domains. Within clade I, two *D. melanogaster *proteins, CG13422 and CG12780, consist of only a signal peptide and CR domain. Both of these *D. melanogaster *paralogues are likely to be functional despite the loss of the GLU domain, as they are upregulated during bacterial infection and CG13422 is also upregulated during fungal infection [27,28]. Clade II contains a species-specific expansion of ten *D. pulex *GNBP paralogues. The only other member of this clade is a GNBP from the oligochaete annelid *Eisenia foetida*, suggesting that insects have lost this GNBP subtype. Many of the *D. pulex *clade II genes are clustered on the same scaffold, and thus probably arose from local duplication events. It is not unusual for diversified duplicated immune genes to be selectively advantageous to new pathogenic challenges [22]. The short branch lengths separating some gene pairs in clade II are indicative of either recent duplication or concerted evolution. None of the GNBP clade II proteins have a CR domain, but all of them, except for Dappu-GNBP1, have an active GLU domain.

GNBP clade III consists of proteins exclusively from crustaceans. However, surprisingly, none of the *D. pulex *GNBP homologues are members, indicating that it is missing from the draft assembly, that it has been lost, or that it diverged substantially since the separation of these species. GNBP clade III proteins have no CR but retain GLU domains. GNBP clade IV consists of five *A. gambiae *proteins and one *D. pulex *protein suggesting an *Anopheles*-specific gene expansion and, in *D. melanogaster *and in other insect species, either loss or substantial divergence. None of the proteins in this clade contains a CR domain and all have an active GLU domain.

### Peptidoglycan recognising proteins (PGRPs)

Another major class of PRR are the peptidoglycan recognition proteins (PGRPs). Aside from recognising peptidoglycan, a required constituent of the cell membranes of gram-negative bacteria, it has been shown in *D. melanogaster *that GNBP1 and PGRP-SA form a complex that leads to the activation of the Toll receptor, resulting in the production of antimicrobial peptides. PGRPs have been implicated in a variety of other immune functions, notably the induction of phagocytosis, activation of the Imd and prophenoloxidase pathways, and may even have direct cytotoxic activity towards bacteria [29]. Surprisingly, our search of the *D. pulex *genome found no PGRP genes. Notably, however, *D. melanogaster *PGRP-LD is part of a complex transcriptional unit that undergoes differential splicing and translation in a different reading frame to yield two very different peptides: PGRP-LD or PMI. Although we recovered a homologue of PMI in *D. pulex*, its alternative reading frames do not encode a PGRP. This is a surprising finding because *D. pulex *has homologues of Toll-1, the receptor that is activated in response to PGRPs' recognition of gram-negative bacteria. Our finding that *D. pulex *has an expansion of GNBP genes relative to *D. melanogaster *could indicate that some GNBPs compensate for the absence of a PGRP.

### The TOLL pathway

The Toll family cell surface receptors play an important role in the innate immune system of both invertebrates and vertebrates. These ancient proteins function as signal transducers, inducing pathways that result in the production of antibacterial and antifungal proteins following the recognition of pathogens via peptidoglycan recognition proteins (PGRPs) and gram-negative binding proteins (GNBPs). Different Toll proteins have specific interactions with particular pathogens [30]. Unlike mammalian Toll-like proteins, which function solely as recognition and signalling proteins resulting in the activation of immune system pathways, some *D. melanogaster *Toll genes are also involved in developmental regulation.

The Toll gene family is among the best characterised in the immune system. All members encode transmembrane proteins that contain signal peptides, leucine-rich repeat (LRR) regions interspersed with cysteine rich areas, and an intracellular C-terminal Toll-interleukin 1 receptor (TIR) domain. LRRs are 22–28 amino acids long and are typically involved in protein-protein interactions. The intracellular TIR domain is involved in signalling and interactions with the other players in the Toll pathway. Signal transduction in the Toll pathway involves three well conserved single copy genes (Tube, Pelle and MyD88), orthologues of two of which, Pelle and MyD88, we have identified in the *D. pulex *genome (Table 1), indicating that this pathway is likely to function in a manner similar to that of *D. melanogaster*.

**Figure 2:**
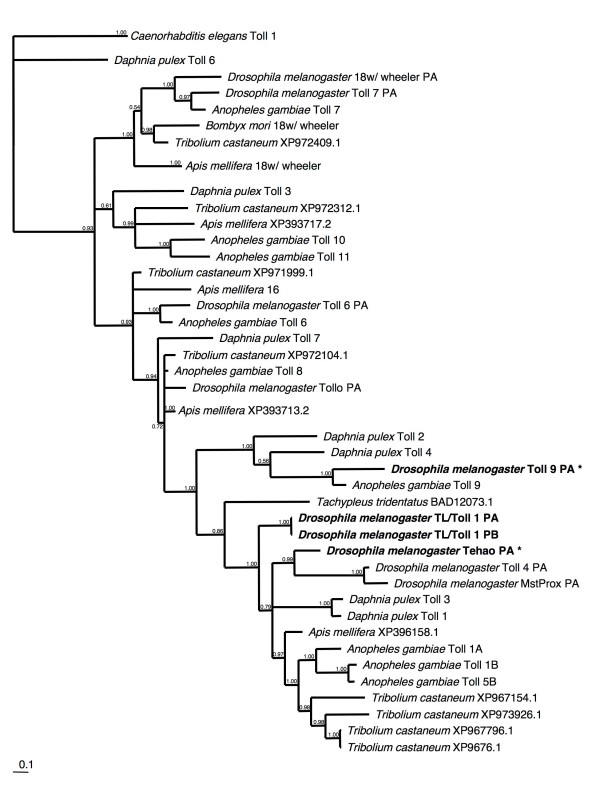
**Bayesian phylogenetic analysis of Toll genes from insects and crustaceans, with *C. elegans *as an outgroup**. All Toll genes thought to be involved in the immune system are highlighted in bold text. Genes marked with * need further *in vivo *experiments to prove their role in immunity. Branch numbers are posterior probabilities.

We found seven Toll receptor genes in the *D. pulex *genome, located on seven different scaffolds (Figure 2). These proteins possess 13 to 28 LRRs, similar to the Toll receptors of *D. melanogaster *(which have from 10–31 LRRs). The variation in the number of LRR repeats makes credible sequence alignment of the full protein sequences problematic, and thus only the relatively conserved TIR domains were used in the phylogenetic analysis (Figure 2). The deep branches of the TIR tree are not well supported, and so it is not possible to comment on the ancestral relationships among the different paralogous groups. However, some of the more recent nodes of the tree are strongly supported, allowing the identification of several candidate immune-related *D. pulex *Toll genes. Dappu-TOLL 1 and Dappu-TOLL 3 are members of a clade that includes *Dm*-Toll1, shown to act as signal transducer for the induction of the Toll pathway following infection with fungi and bacteria [31,32]), and *Dm*-Tehao, implicated in induction of drosomycin in response to fungal infection [33,34]. Dappu-TOLL2 and Dappu-TOLL4 are clustered with *Dm*-Toll-9, which is most similar to the mammalian Toll-like receptors that are involved in signalling to initiate acute inflammatory responses, phagocytosis and antimicrobial peptides. However, definitive evidence of the involvement of *Dm*-Toll-9 in immune responses is lacking. Dappu-TOLL3 differs from Dappu-TOLL4 and the other members of this clade in being encoded by a single, large exon (rather than 6 or 7 exons). This gene may be the result of an ancient duplication mediated by retrotransposition of a mature mRNA.

### Thioester proteins

Invertebrate thioester proteins (TEPs) are an ancient family related to vertebrate complement factors. Most of these proteins contain a thioester (TE) motif (GCGEQ) accompanied by a catalytic histidine residue. These two elements covalently bind pathogens through a thioester bond until the pathogens are cleared by phagocytosis [35]. However, not all TEPs contain a TE motif or catalytic histidine. How TEPs function in the absence of these motifs is currently unknown, but it has been suggested they may act as adaptors for the initiation of the membrane attack complex as is found in vertebrates [36].

Among the best-studied TEP family members are the α_2_-macroglobulins (α_2_m), protease inhibitors that are activated by pathogen-released proteases. This class of TEPs contain a TE motif, but lack a catalytic histidine residue. Upon activation, α_2_m proteins undergo a conformational change that traps the pathogen protease within the protein, leading to the exposure of the TEP C-terminal recognition domain, which binds phagocytic cells and promotes endocytotic clearance.

While not all invertebrate TEPs have a documented immune function, several functional studies have begun to elucidate their role in this capacity. For example, the expression of the *Ag*-TEP1 gene is upregulated after immune challenge and, upon activation, it has been shown to promote phagocytosis [37]. Additionally, some *D. melanogaster *TEPs, Tep1, Tep2 and Tep4 are upregulated following immune challenge [38]. Finally, it has been suggested that different TEP family members bind different pathogens [39].

**Figure 3:**
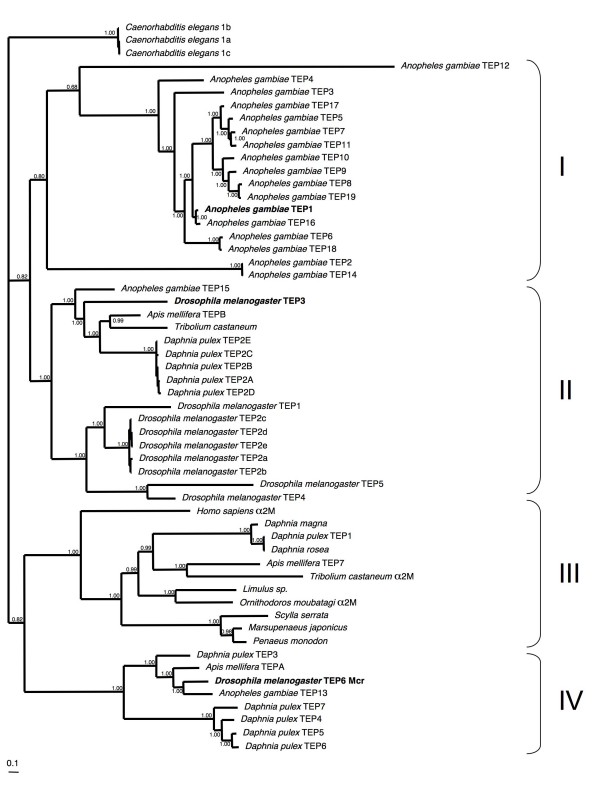
**Bayesian phylogenetic analysis of thioester proteins**. Clades I–IV represent (I) an *Anopheles gambiae *specific species-expansion, (II) all genes except *D. melanogaster *TEP-5 have a TE motif (III) alpha-2-macroglobulin-like genes and (IV) genes lacking a thioester motif (TE). Those genes that have been functionally characterized to play a role in the immune system are highlighted in bold text. Branch numbers are posterior probabilities.

We found seven TEP genes in the *D. pulex *genome. Three are on different scaffolds and four lie clustered on a single scaffold. All seven *D. pulex *TEP proteins have a signal peptide, indicating they are secreted. Our phylogenetic analysis (Figure 3) identified four well-supported clades. TEP clade III unites the mammalian α_2_m with non-vertebrate TEPs including Dappu-TEP1; this clade does not have representatives from *A. gambiae *or *D. melanogaster*. TEP clades II and IV contain 1 and 5 representatives respectively from *D. pulex *and correspond to the previously defined invertebrate TEP class. All members of TEP clade II except *Dm*-TEP5, and all members of clade IV have a TE motif. TEP clade IV includes the *D. melanogaster *macroglobulin complement-related (Mcr) gene, essential for the specific phagocytosis of the fungus *Candida albicans *[39]. Four of the five *D. pulex *proteins in this clade (Dappu-TEP4, -5, -6 and -7) are similar in sequence and are neighbours on one scaffold, and thus probably arose through recent local duplication events. Clade I is an *A. gambiae*-specific expansion.

Regarding the possible immune function of these TEPs, *Dm*-TEP3 from clade II has been shown to act as an opsonin for *Staphylococcus aureus*. Another *D. melanogaster *gene in this clade, *Dm*-TEP2, has five splice variants, the functional significance of which remains to be determined.*Dappu*-TEP2, also in clade II, may also exhibit alternative splice forms as there are additional putative coding exons in the region of the gene homologous to those found to be alternatively spliced in *D. melanogaster*. Interestingly, a clade III TEP from the tick *Ornithodoros moubata *also shows splice variants [40]. Multiple splice forms may serve to increase the repertoire of proteases that are recognised by these TEP proteins. Of the seven *D. pulex *TEP genes, only Dappu-TEP2 has both the TE motif and a catalytic histidine residue suggesting that it likely functions as an opsonin. TEP clade III includes representatives from human and arthropods: from insects (a hymenopteran and a colepteran), two chelicerates, three malacostracan crustaceans and three species of *Daphnia*. As is the case for all the proteins in this clade, Dappu-TEP1 has a TE motif but no histidine residue, and likely functions similarly to the functionally characterized α_2_m molecules.

### Scavenger receptors

Scavenger receptors (SR) are a diverse, multigene family of cell surface membrane proteins that share broad structural similarities. SR recognize and bind modified low-density lipoprotein (LDL), multiple polyanionic ligands and cell wall components [41]. SR have a dual cellular role: they are PRR of the immune system, whose triggering results in the cellular encapsulation of bacteria, and also have a housekeeping role of 'scavenging' cellular debris. Structurally, SR can display different numbers and types of protein domains, including chitin-binding domains, scavenger cysteine-rich receptors (SRCR), low-density lipoproteins (LDL), and C-type lectins (see Additional file 1). SRCR domains are candidates for ligand binding and protein interaction, although their precise biological functions remain largely unknown. We examined only one of three classes of SR, namely the macrophage scavenger receptor class A (SR-A), as they appear to function primarily in the immune system [42,43]. The SR-A class can be divided into two groups: those that have at least one SRCR domain and those that do not (SR-A1 and SR-A2 respectively; see Additional file 1).

**Figure 4:**
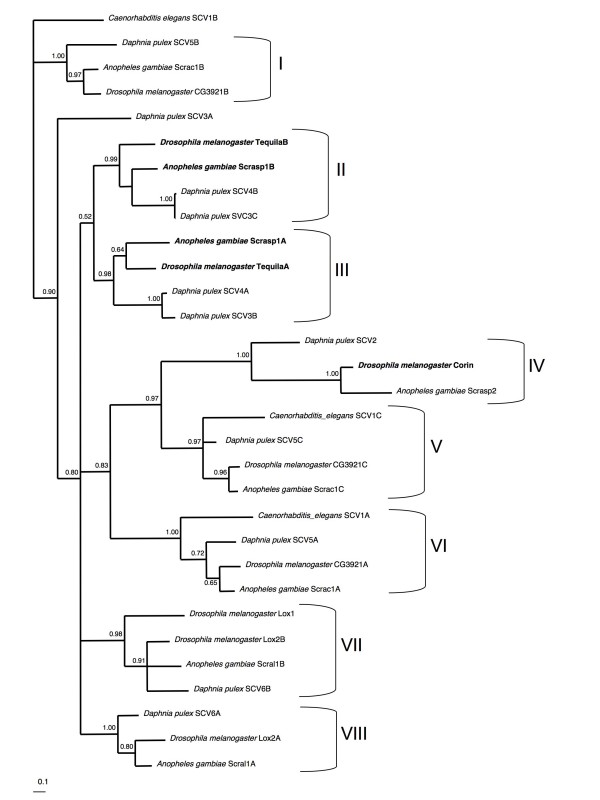
**Bayesian phylogenetic analysis of scavenger receptor domains (SRCR) from scavenger genes from available arthropod sequences**. SRCR copy number varies from 1–3 among the genes analysed. For those genes containing more than one SRCR domain, the gene name has been appended with A, B or C according to the 5' to 3' position of the SRCR domain. Posterior probabilities are indicated at nodes. Genes that have been functionally characterized as members of the immune system repertoire are indicated with bold text.

*D. melanogaster*, *A. gambiae *and *A. aegypti *each have five SR-A genes that form four orthologue pairs [22]. In contrast, six class A scavenger genes were recovered from 6 different scaffolds within the *D. pulex *genome. Structurally, only *A. gambiae *Scrasp3 and Dappu-SCV1 do not contain SRCR domains and are therefore classified as SR-A2 type. Due to the highly variable domain structure of the SR, an alignment of the full protein sequences was not possible. Therefore, we conducted a phylogenetic analysis of the conserved SRCR domains of the remaining fourteen SR-A1 proteins (containing 31 domains) from *D. melanogaster*, *A. gambiae *and *D. pulex*. Thus, a single protein that contains three SRCR domains (labelled A, B, C from the N-terminus) has three representations on the tree (Figure 4).

Eight well-supported groups were resolved, each containing at least one SRCR domain from each of *D. melanogaster, A. gambiae *and *D. pulex*. The fact that orthologous trios are recovered when a crustacean is added to the sequences examined strongly supports the hypothesis that these recognition molecules are under strong functional constraint. SCRC domains found within the same protein are placed in different groups, excluding intragenic domain duplication as a mechanism of their evolution. Further supporting this conclusion is the fact the spatial orientation of the multiple domains is maintained across these diverse taxa, including the single protein examined from the nematode *Caenorhabditis elegans*. The single SRCR domain from group 2 proteins is most similar in sequence to the third SRCR domain from group 4 proteins, but other inter-group relationships were not resolved. This may be due to a rapid burst of domain duplication in an ancestral genome and subsequent maintenance of functionally divergent paralogues.

With regard to the different functional roles of the SR paralogues, the *D. melanogaster *gene encoding the protein Tequila/Graal, a secreted protein that is primarily transcribed in the fat body, was found to be significantly upregulated after immune challenge [44]. Likewise, its orthologue in *A. gambiae*, Scrasp1, is also upregulated after microbial challenge [45,46]. However, disruption of Tequila did not effect the survival of flies infected with either of gram-positive or gram-negative bacteria, the production of antimicrobial peptides or prophenoloxidase activity [44]. Therefore, the role, if any, in immunity of these genes is currently unclear, although it is evident from the experiments with *D. melanogaster *that they do not play a role in the activation of the Toll pathway. *D. pulex *has two co-orthologues of Tequila/Graal/Scrasp1 (Dappu-SCV3 and Dappu-SCV4), both of which are therefore also putatively involved in the immune system. This is the only example of two gene copies from one species within a group, indicating that either a gene duplication has occurred in *D. pulex*, or a gene copy has been lost in the other species. Contrary to what one would expect as the result of recent gene duplication, the two *D. pulex *genes are not identical in domain structure: one (Dappu-SCV3) contains three SRCR domains and two chitin-binding domains, while the other (Dappu-SCV4) contains two SRCR domains and no chitin-binding domains. Similar to Dappu-SCV3, *D. melanogaster *Tequila and *A. gambiae *Scrasp1 both contain at least 2 chitin binding domains, but resemble Dappu-SCV4 in that they each have only two SCRC domains. Nevertheless, a *D. pulex *specific gene duplication seems likely.

The phylogenetic analysis shows that SRCR_B and SCRC_C from Dappu-SCV3 share a common ancestry with the first and second domain copies respectively of Tequila, Scrasp1 and Dappu-SCV4. Thus, Dappu-SCV3 SCRC_A lacks a direct orthologue in the other members of the group, indicating that the other two species may have lost it. Moreover, the branch lengths separating the two *D. pulex *genes are very short. Thus, it appears that in the lineage leading to group 1 proteins in the dipterans have lost a SCRC domain, and that *D. pulex *has undergone a recent species-specific gene duplication resulting in a truncated gene copy with only two SRCR receptors and no chitin-binding domains. Finally, the *D. melanogaster *protein Corin, containing a single SCRC domain, also has a documented putative functional role in the immune system. Indeed, it is up-regulated three-fold when challenged by any of gram-positive, gram-negative or fungal pathogen challenges [28]. Thus, based on our phylogenetic analysis, the Corin orthologue Dappu-SCV2 is likely to have an immune function.

### Chitinases

Chitin is a polysaccharide found in the supportive structures of many organisms including the exoskeletons of invertebrates, cell walls of some fungal spores, and cysts of amoeboid parasites. Chitinases hydrolyse chitin, and are critical in arthropod development (e.g. during ecdysis). A wide range of organisms that do not synthesise chitin also express chitinases where they play roles in chitin digestion and in immune defence against chitin-containing pathogens. For example, plant class I chitinase has been shown to undergo rapid adaptive evolution in its active site cleft, presumably due to an arms race with a fungal pathogen [47]. Additionally, two chitinase-like proteins in *A. gambiae *(*Ag*-AgBr1 and *Ag*-AgBr2) were shown to be upregulated in the presence of gram-positive bacteria [48], and chitinase-like proteins are part of the mammalian immune system [49]. We identified 17 chitinase genes in *D. pulex*, a similar repertoire to that of *D. melanogaster*, which has 16. Intrachromosomal tandem duplication has likely contributed to the large number of chitinase genes within the *D. pulex *genome.

Chitinase and chitinase-like proteins contain two primary structural domains: the glycosyl hydrolase family 18 domain (GH18), which is responsible for hydrolysing chitin oligosacchirides, and the chitin-binding domain (CH14). The number and spatial arrangement of these structural domains varies among the available arthropod sequences. However, all contain between 1 and 5 GH18 domains while only a subset contain CH14 domains.

**Figure 5:**
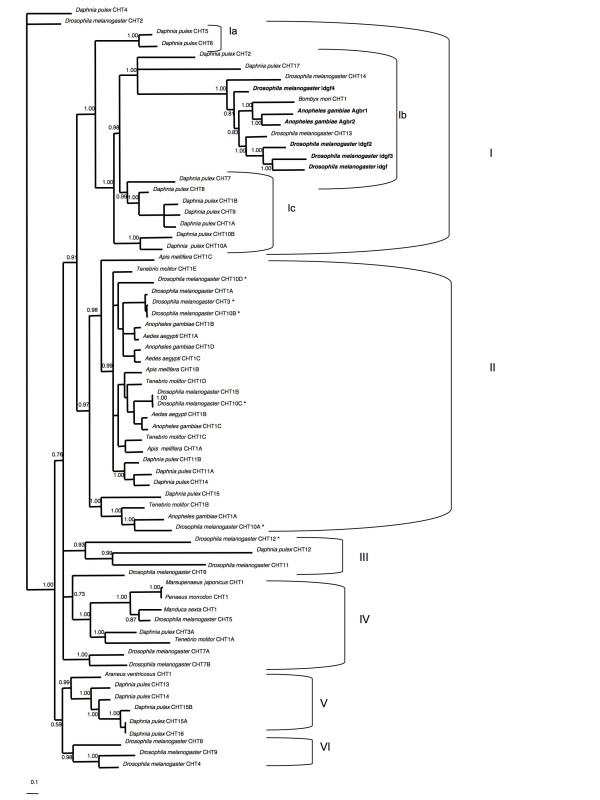
**Bayesian phylogenetic analysis of the glycosyl hydolase family 18 (GH18) domain from arthropod chitinase genes sequences**. The number of GH18 domains in the chitinase genes analysed ranges from 1 to 5. For those genes containing more than one GH18 domain, they are labelled from 5' to 3' with A, B, C, D or E. Clade Ib consists of members that, based on sequence analysis, do not have the ability to hydrolyse chitin. Additional gene copies that cannot hydrolyse chitin are indicated with an asterisks (*). All gene members known to participate in some immune function are indicated in bold text.

All of the chitinase and chitinase-like proteins that have a characterised immune related function contain a single GH18 domain and do not contain a CH14 domain. Phylogenetic analysis of the individual GH18 domains for all available chitinase and chitinase-like genes from various arthropod taxa yielded a well-supported tree (Figure 5), with all the immune-related genes in one distinct clade (clade Ib). The GH18 domains from Dappu-CHT1 and Dappu-CHT2 are closely related to this cluster, and define these two as likely immune-related chitinase genes. Furthermore, all members of clade Ib, including Dappu-CHT1 and Dappu-CHT2, lack an active site glutamate residue critical for the hydrolysis of chitin, a trait that does not appear to be necessary in immune system function. Additionally, based on the longer branch lengths in clade Ib, its members appear to be evolving more rapidly than most of the other clades, a trait consistent with genes experiencing changing selective pressure, such as can be caused by host-pathogen interactions. Interestingly, a further four GH18 domains found elsewhere in the tree also lack the glutamate residue (*Dm*-CHT12, *Dm*-CHT10, Dappu-CHT3, and *Tenebrio molitor *CHT1), indicating that these domains also have a function that does not require hydrolysis of chitin, and should be considered for functional studies.

An additional nine GH18 domains from seven *D. pulex *chitinases are contained within clade I, representing a large species-specific gene expansion. Although all of these gene copies are predicted to have the ability to hydrolyse chitin, such a large gene expansion of genes in the class that may have given rise to the immune-related chitinases warrants functional characterization.

### Nitric oxide synthase (NOS)

The nitric oxide synthase (NOS) genes encode an enzyme responsible for the production of nitric oxide (NO), a highly reactive free radical gas. NO is toxic to nearly all types of pathogens. All vertebrates studied to date have three NOS paralogues, permitting partitioning of gene function. Two paralogues produce constitutively expressed proteins (neuronal and endothelial NOS), while the third, inducible NOS, is an immune response molecule triggered by pro-inflammatory cytokines. In contrast to the vertebrate NOS genes, all previously investigated invertebrates, which include six insect species and one crustacean (the blackback land crab *Gecarcinus lateralis*), have a single NOS that performs multiple metabolic functions, including roles in both humoral and cellular innate immune responses [50-52]. In contrast, we identified two NOS paralogues in the *D. pulex *genome. Dappu-NOS1 and Dappu-NOS2 differ in gene structure, suggesting that they are not the result of a recent duplication event. Indeed, the two *D. pulex *NOS proteins are only 44% identical, and thus are less similar to each other than are mouse inducible NOS and either mouse neuronal NOS (51%) or mouse endothelial NOS (52%).

Our reconstruction of the NOS phylogeny identifies an insect-specific clade with the NOS of the blackback land crab as the sister to this clade (see Additional file 2). However, the relationship of the two *D. pulex *paralogues to this clade is not resolved. Regardless of their evolutionary relationship with the other NOS homologues, the duplication of NOS in *D. pulex *could have resulted in gene subfunctionalization [53], potentially in a manner similar to the NOS genes in vertebrates, neofunctionalization [54], or even a combination of the two (subneofunctionalization [55]). Without further functional information with respect to the product of these two genes, it is not possible to test between these different models. However, the fact the branch lengths of both of the *D. pulex *NOS genes (especially that of Dappu-NOS2) are long relative to other branches within the tree suggests that both of these genes are either experiencing release from selective constraint or are subject to positive selection that has accelerated their evolution.

### Caspases

Caspases are members of the cysteinyl aspartate proteinase family that cleave particular substrates after aspartic acid residues. Caspase proteins contain three domains: prodomain, p20 and p10. The prodomain varies in sequence length and composition, and contains motifs that direct the protein to particular complexes or organelles. The p20 and p10 units are necessary for substrate recognition and catalytic activity.

Members of the caspase family play roles in programmed cell death and inflammation in vertebrates, but their roles in invertebrates are less clear. A phylogenetic analysis of caspase p-domains from *D. melanogaster*, *Danio rerio*, *Xenopus laevis*, *Gallus gallus*, *Mus musculus *and *Homo sapiens *identified three main clades corresponding to inflammatory, apoptotic or apoptotic initiator responses [56]. No arthropod caspase homologues were placed in the clades containing vertebrate inflammation responsive homologues. However, the *D. melanogaster *caspase Dredd mediates immune responses to infection by gram-negative bacteria, possibly by cleaving the antimicrobial peptide transcription factor Relish [57]. Furthermore, a study of the *D. melanogaster *caspases Decay, Daydream and Drice found that all three were upregulated by pathogens [28]. Thus it appears that vertebrate and arthropod caspases may have independently evolved an immune function.

**Figure 6:**
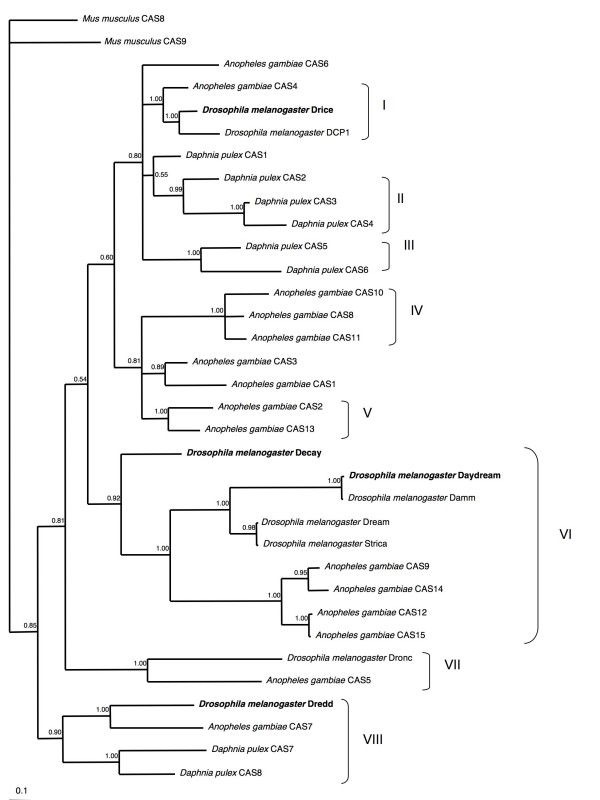
**Bayesian phylogenetic analysis of caspase genes from available insect and crustacean sequences**. Genes with known immune function are highlighted in bold text. Numbers at nodes are posterior probabilities.

We identified eight putative caspases in *D. pulex*, the same number found in *D. melanogaster *and *T. castaneum*. *A. gambiae *has fifteen (including two caspase-like genes), while *A. mellifera *has only one (Table 1). *D. pulex *caspases are distributed among five scaffolds, with three paralogues arranged on a single scaffold. The prodomain of the various caspases was too variable to align with confidence and thus was excluded from further analysis. In contrast, the p20 and p10 domains were sufficiently similar to allow an alignment to be constructed. The phylogenetic tree of the caspases contains 8 clades, the relationship among which is unresolved (Figure 6). There are only two 1:1 orthologues between *D. melanogaster *and *A. gambiae*, and neither orthologous pair includes a *D. pulex *member. However, all but one *D. pulex *and one *A. gambiae *caspase cluster with paralogues from the same species, indicating that lineage-specific gene duplication is a relatively frequent event.

Two of the eight clades (clades II and III), containing a total five genes, are unique to *D. pulex*, while another (clade IV) is unique to *A. gambiae*. Only clade VIII, containing the immune related *Dm*-Dredd, includes orthologues from all three arthropods, suggesting that the putative immune function of this clade is ancestral to the split between the crustaceans and insects. The transcription factor Relish that is activated by Dredd is also conserved among these species. However, only *D. pulex *contains two paralogues within clade VIII, a lineage-specific expansion that may provide additional flexibility in immune-related function. The other caspases with characterized immune function in insects are found in clades I and V, neither of which contains *D. pulex *orthologues.

### Anitviral RNAi genes

RNA interference (RNAi) is an ancient defence mechanism that targets invading viral double-stranded RNA and transposons [58]. The RNAi cascade involves many genes working in synchrony, including Argonaute, an endonuclease, and Dicer, which is responsible for cutting dsRNA into small fragments for further processing. It has been shown that genes involved in the RNAi pathway are evolving rapidly due to positive selection, and that they show patterns of nucleotide polymorphisms that are consistent with a recent selective sweep [5]. Based on these findings, it is suggested that the rapid adaptive evolution in these genes may be caused by a coevolutionary arms race between viral pathogens and host defence [5].

**Figure 7:**
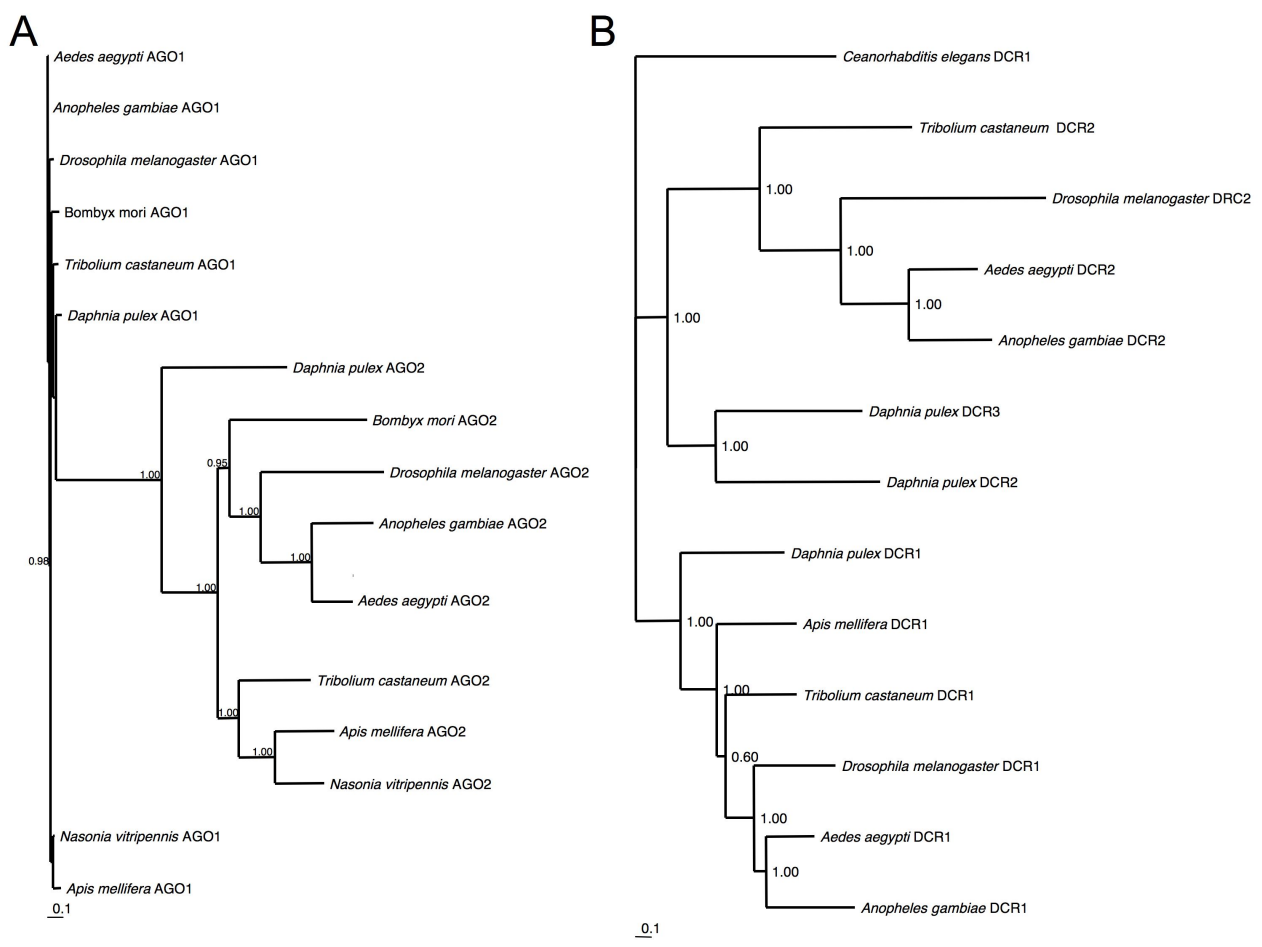
**Bayesian phylogenetic analysis of two RNAi genes**. **(A) **Argonaut genes, and **(B) **Dicer genes. Based on functional studies in *Drosophila*, AGO1 and DCR1 gene copies are thought to play a housekeeping function, whilst AGO2 and DCR2 gene copies are thought to be involved in antiviral pathways. Numbers at nodes are Bayesian posterior probabilities.

As expected from the *D. melanogaster *genome, we identified two Argonaute genes in *D. pulex*, corresponding a housekeeping endonuclease with no immune function, and an RNAi endonuclease (Figure 7a). Contrary to expectations from *D. melanogaster*, three Dicer paralogues were identified in the *D. pulex *genome. Dicer paralogues resolve into two primary clusters corresponding to housekeeping genes and those that take part in the antiviral activity (Figure 7b). The additional *D. pulex *Dicer gene appears to be the result of a lineage-specific duplication of the antiviral pathway paralogue. These duplicates show lower sequence similarity to each other than do the orthologues from the mosquitoes *Aedes aegypti *and *A. gambiae*, so the duplication event is unlikely to be new. From their respective branch lengths, both the *D. pulex *antiviral Dicer proteins are evolving at a higher rate than the housekeeping paralogues, a finding consistent with the hypothesis that they are undergoing a co-evolutionary arms race with *D. pulex *pathogens. Similarly, the *D. pulex *Argonaute gene copy in the putative antiviral pathway shows a higher rate of evolution than the housekeeping paralogue. A population genetic survey could test whether these genes are indeed experiencing strong selective pressures.

### Immunoglobulin-related

In vertebrates, the innate immune system is complemented by a complex adaptive immune system that generates novelty in immune receptors by somatic recombination and rearrangement of immunoglobulin-domain containing genes. While there has been no evidence that non-vertebrates have homologous machinery for an adaptive immune response, one gene with an immunoglobulin domain, a homologue of the human Down Syndrome cell adhesion molecule (DSCAM), has recently been implicated in immune-related somatic diversification in insects [59,60]. DSCAM is a key player controlling neural wiring in both vertebrates and invertebrates. However, while vertebrates can make three different transcripts through alternative splicing, *D. melanogaster *has the capacity to make 30,000 unique transcripts due to four clusters of variable exons spliced in a mutually exclusive manner. This remarkable gene structure has been observed in all insects studied to date, including the dipterans *D. melanogaster*, *A. gambiae*, and *A. aegypti*, the lepidopteran *Bombyx mori*, the hymenopteran *A. mellifera*, and the colepteran *T. castaneum*, although alternative exon number is variable.

It has been speculated that the alternative transcripts may yield protein isoforms that act in a manner similar to that of vertebrate antibodies. Dscam transcripts have been found in fly hemolymph, fat body cells and hemocytes [60]. In these tissues, novel alternative splice products were found that have not been observed in neural tissue. Furthermore, *A. gambiae *challenged with different types of pathogens resulted in the transcription of different Dscam variants [59]. RNAi experiments in both the fly and mosquito have shown that the organisms were less able to clear pathogens when Dscam was inhibited [59,60]. Dappu-Dscam, fully described in a companion paper [61], has similar complexity to that observed in insects (Dappu-Dscam contains three variable exon clusters) and the frequency of alternative transcript expression differs between the brain and hemocytes, suggesting that *Daphnia *DSCAM may also play a role in immunity.

## Conclusion

Natural populations vary tremendously in their levels of parasitism and pathogen virulence, and experimental investigations of infection phenotypes have revealed that host genetic polymorphism often mediates these patterns. However, it is rarely known which genes underlie this variation. *Daphnia *is a prime example of this, as much is known about its phenotypic responses to pathogens, both in the field and in the laboratory, but its immune system is relatively unknown. Thus, in addition to the phylogenetic insights gained in the present study, annotating the *Daphnia *immune-related genome raises the possibility of studies that link genetic variation to ecosystem dynamics. This is a key advance relative to *Drosophila*, which although genetically well characterised and the source of exciting insight into innate immunity, is less well studied in its natural environment.

The bioinformatics approach used in this study provides the necessary first step in understanding the *Daphnia *immune system. However, as it relies on searching for genes that are characterized in other organisms, it does not provide a complete picture. We will not be able to gain a full understanding of the *Daphnia *immune system until we identify those immunity genes that are unique to *Daphnia*, for example by looking at the transcriptome of infected versus healthy individuals. Furthermore, most work on natural variation in responses to *Daphnia *pathogens has been carried out on *D. magna*, while the current genome sequence is for the rather distantly related *D. pulex*. Nevertheless, we have so far had substantial success transferring genomic information from *D. pulex *to *D. magna *(for example, designing oligonucleotide primers useful for accessing the *D. magna *transcriptome). Moreover, the *D. pulex *genome will prove a useful resource for characterising the *D. magna *genome, which is an ongoing sequencing project undertaken by the *Daphnia Genomics Consortium*. Thus with the immune related genome of *Daphnia pulex *annotated, development of a system for in-depth ecological genetics of infection is now within our grasp.

## Methods

### *In silico *identification of *D. pulex *immune genes

We conducted all gene searches on the *D. pulex *8.7× coverage scaffold assembly (Dappu v1.1, September 2006;  and ). Each search was initiated by identifying all arthropod protein sequence homologues of the target gene from GenBank (see Additional File 3 for accession numbers of all publicly available genes shown in the phylogentic trees, and Additional File 4 for sequences of gene models constructed from publicly available genome trace files). These were then compared to the 5,191 scaffolds using tBLASTn, and all high scoring segment pairs with an E-value of less than 10^-5 ^selected. The regions of the scaffolds corresponding to significant matches, plus an additional 2000 bases up- and down-stream, were extracted as a single unit using a Perl script. The genome annotation workbench Artemis  was used to identify and annotate probable gene structures in *D. pulex *using additional information deriving from BLASTn searches of the cognate *D. pulex *expressed sequence tag dataset, BLASTp searches of the open reading frames of the target scaffold sequence against UniProt, % GC content, and GT-AG intron boundaries.

The protein domain structure of each amino acid sequence was inferred using the Pfam website . Signal peptides and transmembrane regions were identified using the SignalP server . To search for additional matches that might indicate the presence of gene families, each annotated gene was used to interrogate the *D. pulex *genome assembly. This entire procedure was carried out until no new gene copies were identified. The sequences of the *D. pulex *gene models are available in Additional file 5.

### Gene nomenclature

Newly identified genes were named according to the recommendation of the *Daphnia *Genomics Consortium . Briefly, an uppercase three-letter symbol is assigned to each gene, followed by a number when the gene is orthologous to a *D. melanogaster *gene, and subsequently followed by an uppercase letter if the numbered gene has paralogues in *D. pulex*. For cases where the genes annotated had a previously accepted nomenclature, we followed the extant naming convention, even if it deviated from the three-letter code (e.g. gram-negative binding proteins are annotated as GNBP), although the convention for assignment of homology to insects and different loci in *D. pulex *was still adopted. In the text, *D. pulex *genes are identified by the prefix Dappu, those of *D. melanogaster *by prefix *Dm*, and those of *A. gambiae *by *Ag*.

### Phylogenetic inference

The *D. pulex *protein sequences were aligned to homologous sequences from other taxa using the ClustalW algorithm within MacVector (v7.2.3). Multiple sequence alignments were corrected by eye. In some cases, these alignments were used to refine the *D. pulex *gene annotation, for example when a highly divergent exon was difficult to identify by BLAST score and % GC content alone. The completed multiple sequence alignment was used to infer a phylogeny using MrBayes 3.1.2. We used the mixed model option to choose the amino acid substitution model, a gamma rate distribution parameter estimated from our dataset, and saving every 100^th ^tree. Two parallel Markov chains were run simultaneously in each of two runs. Tree length, amino acid model, log-likelihood score and alpha value of the gamma distribution were examined in the program Tracer v1.3 prior to the termination of MrBayes to ensure that all parameters had reached stationarity. Saved trees from after the burn-in were summarised and posterior probabilities estimated.

### Gene amplification from cDNA

When the validity of a predicted gene was in doubt, we amplified a portion of the gene in question from cDNA (carried out for NOS1, NOS2, Pelle, PPO, GNBP3, GNBP11, TEP1). To this end, we extracted RNA from five wild-caught *D. pulex *that were visibly infected with microsporidia and stored in RNA-later (Quiagen Inc) using the following methods. Samples were pooled by centrifugation at 12000 rpm for 1 minute, and the RNA-later was removed. Trizol (Gibco BRL) was added to the remaining *Daphnia*, which were subsequently crushed with a homogenizer. Total RNA was extracted following the manufacturer's protocol for phase separation, precipitation and wash steps. An additional DNase I (Ambion) step was performed according to the manufacturer's instructions. Total RNA was quantified by UV spectophotometry and approximately half of the total RNA was further processed using the Poly(A)purist kit (Ambion) according to the recommended protocol in order to isolate mRNA. Reverse-transcription polymerase chain reaction (RT-PCR) was carried out from both total RNA and mRNA to make cDNA in 10 μl reactions using 250 ng of total RNA or 10 ng of mRNA using the Accuscript high fidelity PCR system (Strategene).

All primers were designed within exons using Primer3 . PCR was carried out in 50 μl reactions using a mix containing 1 μl of cDNA made from either the total RNA or mRNA (used to amplify rare transcripts), and 1 unit of *Pfu *(Strategene) as outlined in the enzyme instructions. In addition, a single pair of primers was designed such that they overlaid intron/exon boundaries of a portion of the *D. pulex *prophenoloxidase gene. These primers were included as a control in a PCR from the total RNA and mRNA in order to verify that no genomic template contaminant was present in the sample. The PCR cycling profile was as follows: 95°C for 1 minute, followed by 39 cycles of 95°C for 30 seconds, 56°C for 30 seconds, 68°C for 3 minutes/kb of product; a final extension of 68°C for 10 minutes completed the PCR program.

Each PCR product was cleaned by treatment of shrimp alkaline phosphotase and exonuclease I. Treated PCR products were sequenced directly in both directions in a 10 μl mix containing 1.0 μl Big Dye (Amersham), 3.0 μl 5× buffer (Amersham), 0.5 μl primer and 1–3 μl of PCR product. Sequences were aligned to the genomic scaffold sequence to verify that the correct gene had been amplified and that the annotation was correct.

## Authors' contributions

CC and SJM annotated the genes, produced the sequence alignments and phylogenetic analyses, and participated in writing the manuscript. MLB participated in the design of the study, assisted in all stages of the bioinformatics and edited the manuscript, TJL conceived of the study, and participated in its design and coordination and helped to draft and refine the manuscript. JKC provided the EST sequences and guidance on aspects of the bioinformatics analyses.

## Supplementary Material

Additional file 1 **Five domain groupings of Scavenger A genes from *D. melanogaster *(Dmel), *A. gambiae *(Agam), *C. elegans *and *D. pulex *(Dpul)**. Abbreviations of domains: chitin-binding domain = cbd, scavenger receptor = SCRC (labelled 5' to 3' as A, B or C within a gene copy), low-density lipoprotein = ldl. Vertical lines indicate homologous SCRC domains as shown in phylogenetic tree. Groups 1–4 are all SR-A1, while group 5 is SR-A2 (see text). Click here for file

Additional file 2 **Bayesian phylogeny of the nitric oxide synthase (NOS) gene from available insect and crustacean sequences, with the three *Mus musculus *NOS paralogues as outgroup sequences.** Numbers at the nodes are posterior probabilities. Click here for file

Additional file 3 **Accession numbers of all publicly available genes mentioned in manuscript.** Click here for file

Additional file 4 **Gene model sequences constructed by Darren Obbard of Argonaute and Dicer in FASTA format.** Click here for file

Additional file 5 **List of *Daphnia pulex *gene models**. For each gene model entry we list the gene name (ID) and coding DNA sequence (SQ). Additionally, under the tag FT, we give the coordinates of the nucleotide sequence to the JGI *Daphnia pulex *genome sequence, version 060905, unless otherwise noted. Click here for file

## References

[B1] Hoffmann JA, Kafatos FC, Janeway CA, Ezekowitz RAB (1999). Phylogenetic perspectives in innate immunity. Science.

[B2] Hurst LD, Smith NGC (1999). Do essential genes evolve slowly?. Current Biology.

[B3] Little TJ, Cobbe N (2005). The evolution of immune-related genes from disease carrying mosquitoes: diversity in a peptidoglycan- and a thioester-recognizing protein. Insect Molecular Biology.

[B4] Little TJ, Colbourne JK, Crease TJ (2004). Molecular evolution of Daphnia immunity genes: Polymorphism in a gram-negative binding protein gene and an alpha-2-macroglobulin gene. Journal of Molecular Evolution.

[B5] Obbard DJ, Jiggins FM, Halligan DL, Little TJ (2006). Natural selection drives extremely rapid evolution in antiviral RNAi genes. Current Biology.

[B6] Schlenke TA, Begun DJ (2003). Natural selection drives drosophila immune system evolution. Genetics.

[B7] Evans JD, Aronstein K, Chen YP, Hetru C, Imler JL, Jiang H, Kanost M, Thompson GJ, Zou Z, Hultmark D (2006). Immune pathways and defence mechanisms in honey bees Apis mellifera. Insect Molecular Biology.

[B8] Zou Z, Evans JD, Lu ZQ, Zhao PC, Williams M, Sumathipala N, Hetru C, Hultmark D, Jiang HB (2007). Comparative genomic analysis of the Tribolium immune system. Genome Biology.

[B9] Carius HJ, Little TJ, Ebert D (2001). Genetic variation in a host-parasite association: Potential for coevolution and frequency-dependent selection. Evolution.

[B10] Chadwick W, Little TJ (2005). A parasite-mediated life-history shift in Daphnia magna. Proceedings of the Royal Society B-Biological Sciences.

[B11] Duncan AB, Little TJ (2007). Parasite-driven genetic change in a natural population of Daphnia. Evolution.

[B12] Little TJ, Killick SC (2007). Evidence for a cost of immunity when the crustacean Daphnia magna is exposed to the bacterial pathogen Pasteuria ramosa. Journal of Animal Ecology.

[B13] Little TJ, Watt K, Ebert D (2006). Parasite-host specificity: Experimental studies on the basis of parasite adaptation. Evolution.

[B14] Mitchell SE, Read AF, Little TJ (2004). The effect of a pathogen epidemic on the genetic structure and reproductive strategy of the crustacean Daphnia magna. Ecology Letters.

[B15] Decaestecker E, Gaba S, Raeymaekers JAM, Stoks R, Van Kerckhoven L, Ebert D, De Meester L (2007). Host-parasite 'Red Queen' dynamics archived in pond sediment. Nature.

[B16] Limburg PA, Weider LJ (2002). 'Ancient' DNA in the resting egg bank of a microcrustacean can serve as a palaeolimnological database. Proceedings of the Royal Society of London Series B-Biological Sciences.

[B17] Weider LJ, Lampert W, Wessels M, Colbourne JK, Limburg P (1997). Long-term genetic shifts in a microcrustacean egg bank associated with anthropogenic changes in the Lake Constance ecosystem. Proceedings of the Royal Society of London Series B-Biological Sciences.

[B18] Christophides GK, Zdobnov E, Barillas-Mury C, Birney E, Blandin S, Blass C, Brey PT, Collins FH, Danielli A, Dimopoulos G (2002). Immunity-related genes and gene families in Anopheles gambiae. Science.

[B19] Du Pasquier L (2001). The immune system of invertebrates and vertebrates. Comparative Biochemistry and Physiology B-Biochemistry & Molecular Biology.

[B20] Janeway CA, Medzhitov R (2002). Innate immune recognition. Annual Review of Immunology.

[B21] Soderhall K, Cerenius L (1998). Role of the prophenoloxidase-activating system in invertebrate immunity. Current Opinion in Immunology.

[B22] Waterhouse RM, Kriventseva EV, Meister S, Xi ZY, Alvarez KS, Bartholomay LC, Barillas-Mury C, Bian GW, Blandin S, Christensen BM (2007). Evolutionary dynamics of immune-related genes and pathways in disease-vector mosquitoes. Science.

[B23] Insect immune-related genes and gene families. http://cegg.unige.ch/Insecta/iinnumodb/.

[B24] BeeBase: The honey bee genome project. http://racerx00.tamu.edu/bee_resources.html.

[B25] BeetleBase: Tribolium genome database. http://beetlebase.org/.

[B26] VectorBase. http://www.vectorbase.org/index/php.

[B27] De Gregorio E, Spellman PT, Rubin GM, Lemaitre B (2001). Genome-wide analysis of the Drosophila immune response by using oligonucleotide microarrays. Proceedings of the National Academy of Sciences of the United States of America.

[B28] Irving P, Troxler L, Heuer TS, Belvin M, Kopczynski C, Reichhart JM, Hoffmann JA, Hetru C (2001). A genome-wide analysis of immune responses in Drosophila. Proceedings of the National Academy of Sciences of the United States of America.

[B29] Hultmark D (2003). Drosophila immunity: paths and patterns. Current Opinion in Immunology.

[B30] Khush RS, Leulier F, Lemaitre B (2002). Immunology: Pathogen surveillance – the flies have it. Science.

[B31] Lemaitre B, Nicolas E, Michaut L, Reichhart JM, Hoffmann JA (1996). The dorsoventral regulatory gene cassette spatzle/Toll/cactus controls the potent antifungal response in Drosophila adults. Cell.

[B32] Michel T, Reichhart JM, Hoffmann JA, Royet J (2001). Drosophila Toll is activated by Gram-positive bacteria through a circulating peptidoglycan recognition protein. Nature.

[B33] Luo CH, Zheng LB (2000). Independent evolution of Toll and related genes in insects and mammals. Immunogenetics.

[B34] Tauszig S, Jouanguy E, Hoffmann JA, Imler JL (2000). Toll-related receptors and the control of antimicrobial peptide expression in Drosophila. Proceedings of the National Academy of Sciences of the United States of America.

[B35] Levashina EA, Moita LF, Blandin S, Vriend G, Lagueux M, Kafatos FC (2001). Conserved role of a complement-like protein in phagocytosis revealed by dsRNA knockout in cultured cells of the mosquito, Anopheles gambiae. Cell.

[B36] Blandin S, Levashina EA (2004). Thioester-containing proteins and insect immunity. Molecular Immunology.

[B37] Blandin S, Shiao SH, Moita LF, Janse CJ, Waters AP, Kafatos FC, Levashina EA (2004). Complement-like protein TEP1 is a determinant of vectorial capacity in the malaria vector Anopheles gambiae. Cell.

[B38] Lagueux M, Perrodou E, Levashina EA, Capovilla M, Hoffmann JA (2000). Constitutive expression of a complement-like protein in Toll and JAK gain-of-function mutants of Drosophila. Proceedings of the National Academy of Sciences of the United States of America.

[B39] Stroschein-Stevenson SL, Foley E, O'Farrell PH, Johnson AD (2006). Identification of Drosophila gene products required for phagocytosis of Candida albicans. Plos Biology.

[B40] Saravanan T, Weise C, Sojka D, Kopacek P (2003). Molecular cloning, structure and bait region splice variants of alpha(2)-macroglobulin from the soft tick Ornithodoros moubata. Insect Biochemistry and Molecular Biology.

[B41] Krieger M, Herz J (1994). Structures and Functions of Multiligand Lipoprotein Receptors – Macrophage Scavenger Receptors and Ldl Receptor-Related Protein (Lrp). Annual Review of Biochemistry.

[B42] Kim S, Watarai M, Suzuki H, Makino S, Kodama T, Shirahata T (2004). Lipid raft microdomains mediate class A scavenger receptor-dependent infection of Brucella abortus. Microbial Pathogenesis.

[B43] Pierini LM (2006). Uptake of serum-opsonized Francisella tularensis by macrophages can be mediated by class A scavenger receptors. Cellular Microbiology.

[B44] Munier AI, Medzhitov R, Janeway CA, Doucet D, Capovilla M, Lagueux M (2004). graal: a Drosophila gene coding for several mosaic serine proteases. Insect Biochemistry and Molecular Biology.

[B45] Danielli A, Loukeris TG, Lagueux M, Muller HM, Richman A, Kafatos FC (2000). A modular chitin-binding protease associated with hemocytes and hemolymph in the mosquito Anopheles gambiae. Proceedings of the National Academy of Sciences of the United States of America.

[B46] Gorman MJ, Andreeva OV, Paskewitz SM (2000). Molecular characterization of five serine protease genes cloned from Anopheles gambiae hemolymph. Insect Biochemistry and Molecular Biology.

[B47] Bishop JG, Dean AM, Mitchell-Olds T (2000). Rapid evolution in plant chitinases: Molecular targets of selection in plant-pathogen coevolution. Proceedings of the National Academy of Sciences of the United States of America.

[B48] Shi L, Paskewitz SM (2004). Identification and molecular characterization of two immune-responsive chitinase-like proteins from Anopheles gambiae. Insect Molecular Biology.

[B49] Nair MG, Gallagher LJ, Taylor MD, Loke P, Coulson PS, Wilson RA, Maizels RM, Allen JE (2005). Chitinase and Fizz family members are a generalized feature of nematode infection with selective Upregulation of Ym1 and F10.1 by antigen-presenting cells. Infection and Immunity.

[B50] Dimopoulos G, Seeley D, Wolf A, Kafatos FC (1998). Malaria infection of the mosquito Anopheles gambiae activates immune-responsive genes during critical transition stages of the parasite life cycle. Embo Journal.

[B51] Imamura M, Yang J, Yamakawa M (2002). cDNA cloning, characterization and gene expression of nitric oxide synthase from the silkworm, Bombyx mori. Insect Molecular Biology.

[B52] Luckhart S, Vodovotz Y, Cui LW, Rosenberg R (1998). The mosquito Anopheles stephensi limits malaria parasite development with inducible synthesis of nitric oxide. Proceedings of the National Academy of Sciences of the United States of America.

[B53] Hughes AL (1994). The Evolution of Functionally Novel Proteins after Gene Duplication. Proceedings of the Royal Society of London Series B-Biological Sciences.

[B54] Ohno S (1970). Evolution by gene duplication.

[B55] He XL, Zhang JZ (2005). Rapid subfunctionalization accompanied by prolonged and substantial neofunctionalization in duplicate gene evolution. Genetics.

[B56] Lamkanfi M, Declercq W, Kalai M, Saelens X, Vandenabeele P (2002). Alice in caspase land. A phylogenetic analysis of caspases from worm to man. Cell Death and Differentiation.

[B57] Leulier F, Rodriguez A, Khush RS, Abrams JM, Lemaitre B (2000). The Drosophila caspase Dredd is required to resist Gram-negative bacterial infection. Embo Reports.

[B58] Li HW, Ding SW (2005). Antiviral silencing in animals. Febs Letters.

[B59] Dong YM, Taylor HE, Dimopoulos G (2006). AgDscam, a hypervariable immunoglobulin domain-containing receptor of the Anopheles gambiae innate immune system. Plos Biology.

[B60] Watson FL, Puttmann-Holgado R, Thomas F, Lamar DL, Hughes M, Kondo M, Rebel VI, Schmucker D (2005). Extensive diversity of Ig-superfamily proteins in the immune system of insects. Science.

[B61] Brites D, McTaggart S, Morris K, J A, Thomas K, Colson I, Fabbro T, Little TJ, Ebert D, Du Pasquier L (2008). The Dscam homologue in *Daphnia *is diversified by alternative splicing like in insects. Molecular Biology and Evolution.

