# Supplementary figures and images for: The components of the Daphnia pulex immune system as revealed by complete genome sequencing
Source: BMC Genomics. 2009 Apr 22;10:175. doi: 10.1186/1471-2164-10-175 (PMC2685406; doi:10.1186/1471-2164-10-175)

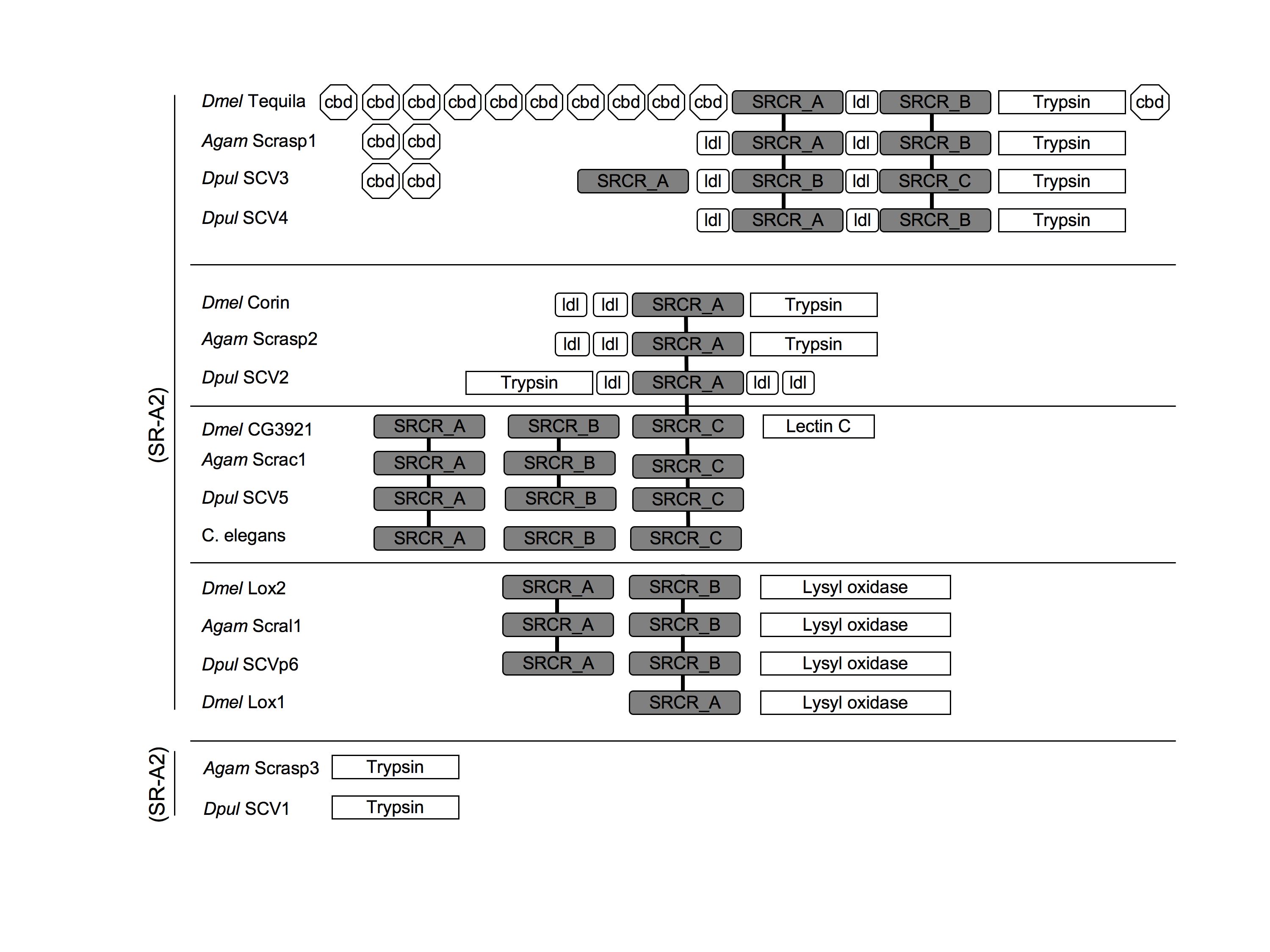

Supplement: Additional file 1 — Five domain groupings of Scavenger A genes from D. melanogaster (Dmel), A. gambiae (Agam), C. elegans and D. pulex (Dpul). Abbreviations of domains: chitin-binding domain = cbd, scavenger receptor = SCRC (labelled 5' to 3' as A, B or C within a gene copy), low-density lipoprotein = ldl. Vertical lines indicate homologous SCRC domains as shown in phylogenetic tree. Groups 1–4 are all SR-A1, while group 5 is SR-A2 (see text). [file 1471-2164-10-175-S1.jpeg]

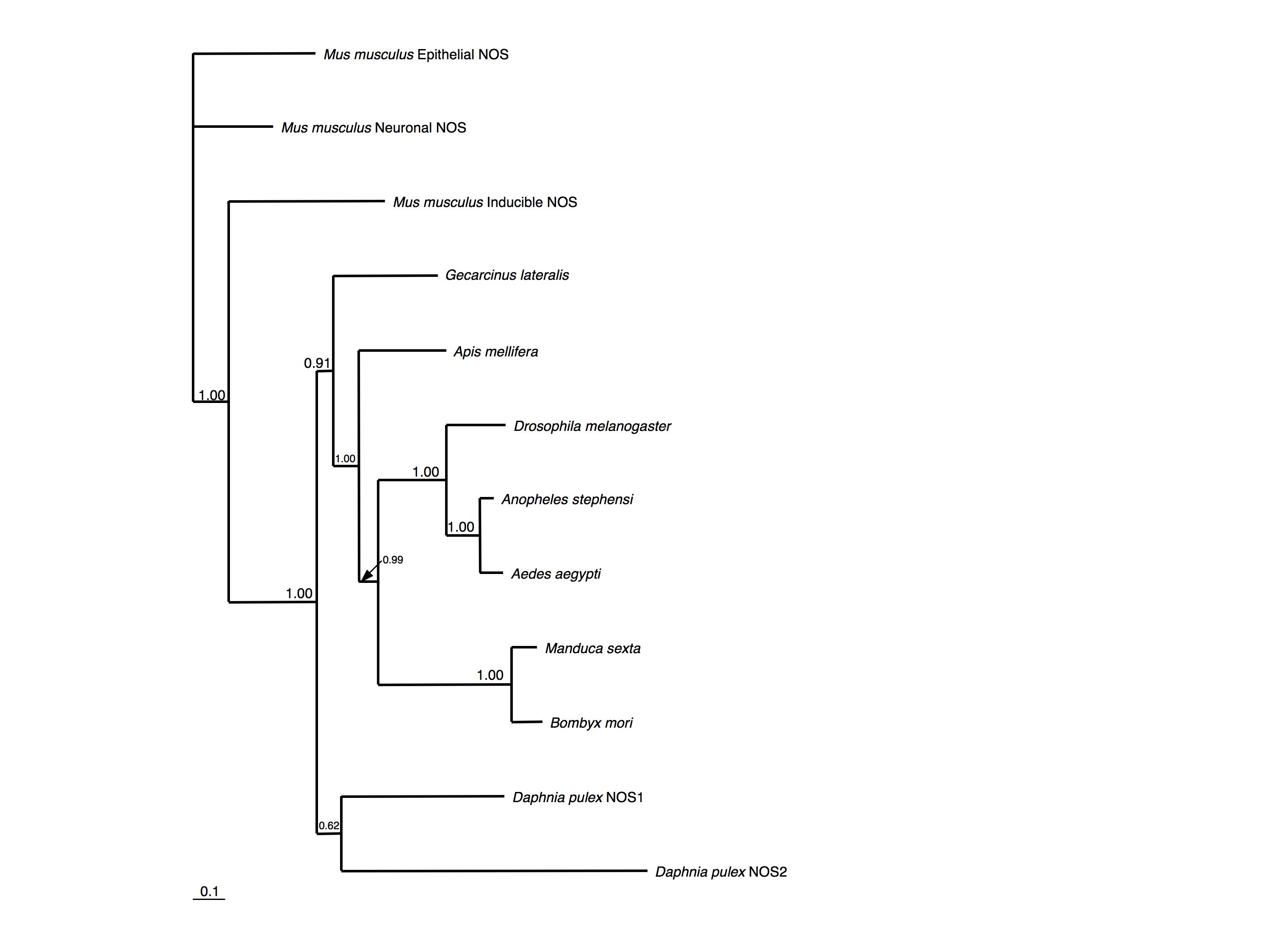

Supplement: Additional file 2 — Bayesian phylogeny of the nitric oxide synthase (NOS) gene from available insect and crustacean sequences, with the three Mus musculus NOS paralogues as outgroup sequences. Numbers at the nodes are posterior probabilities. [file 1471-2164-10-175-S2.jpeg]
